# Designing Safer Chemicals

**DOI:** 10.1289/ehp.1206349

**Published:** 2013-01-01

**Authors:** Linda S. Birnbaum

**Affiliations:** Director, NIEHS and NTP, National Institutes of Health, Department of Health and Human Services, Research Triangle Park, North Carolina, E-mail: birnbaumls@niehs.nih.gov

Many of the > 83,000 chemicals currently used in commerce pose potential toxic hazards to human health and the environment. Numerous policies and systems are in place to try to identify harmful chemicals that are already in use, but removing these problem chemicals once they are already in the marketplace can be difficult, time-consuming, and costly. In an ideal world, chemists would be able to identify problematic chemicals early in the design process—before they are released into the marketplace. This is a central goal of “green chemistry.”

An article recently published in the U.K. Royal Society of Chemistry’s journal *Green Chemistry* (Schug et al. 2012) represents a major step toward realizing the green chemistry ideal. The authors, an esteemed group of green chemists, biologists, and toxicologists, introduce a Tiered Protocol for Endocrine Disruption (TiPED) that chemists can use to detect potentially problematic chemicals during the design process (TiPED 2012).

Endocrine-disrupting compounds (EDCs) are a growing area of concern in toxicology and public health. EDCs can cause significant human health problems because of their ability to mimic common hormones, or interfere with their actions, at very low levels of exposure. Industry and agencies have been slow to develop the analytical tools necessary to address this concern, even as consumer interest has burgeoned in gaining access to products that are, for example, bisphenol A free.

TiPED, which focuses explicitly on detecting the endocrine-disrupting potential of a new chemical early in the design process, will be useful for companies wishing to respond to this consumer interest. However, it is a voluntary approach, not a regulatory program.

To effectively evaluate a chemical’s potential for endocrine disruption, a testing protocol must be able to measure hormone-like effects or perturbations at very low chemical doses, as well as to take into account the many possible ways the chemical may interact with endocrine signaling pathways. The TiPED process starts with a chemist theoretically at “the drawing board” and consists of five testing tiers, ranging from broad *in silico* evaluation through specific cell- and whole organism-based assays; it begins with the quickest and cheapest assays, leading to more specialized tests as needed.

TiPED’s initial two phases rely on predictive computer modeling and high throughput screening to quickly weed out problem chemicals. These tests are followed by more specific *in vitro* cell-based screening assays to refine results and reduce animal testing as much as possible. The final two phases involve the use of fish/amphibian and mammalian *in vivo* models. The screening assays in each tier are based on the most up-to-date science; collectively, the TiPED tiers are designed to cover all known aspects of endocrine disruption.

TiPED is important because, so far, no organized program like it exists in government or in the private sector. The protocol is a promising new development in an effort to help chemists establish a high degree of confidence that the replacements or alternatives they are developing are safe. Because of the high cost of detecting, removing, and replacing toxic chemicals once they are in the marketplace, adopting this protocol can lead to cost savings for companies and government agencies.

In their article, Schug et al. (2012) provide a strong intellectual framework for broadly investigating the endocrine system for signals of endocrine disruption. The authors are currently embarking on a series of field tests with interested companies. Their experience in this next phase will then guide modifications, if needed, and will indicate if and how TiPED can operate in real-world settings. The TiPED approach is a scientifically sound and creative way to chart the course toward a new generation of materials that are inherently safer and also potentially provide financial rewards for chemists and companies that use it.
